# Two mixed-valent cerium oxo clusters: synthesis, structure, and self-assembly

**DOI:** 10.3389/fchem.2024.1507834

**Published:** 2024-12-02

**Authors:** Yuan Gao, Yang Zhang, Zhe Han, Chunhui Wang, Lei Zhang, Jie Qiu

**Affiliations:** ^1^ School of Energy and Power Engineering, Xi’an Jiaotong University, Xi’an, China; ^2^ Engineering Laboratory of Advanced Energy Materials, Ningbo Institute of Materials Technology and Engineering, Chinese Academy of Sciences, Ningbo, China

**Keywords:** cerium oxo cluster, mixed-valent, redox reaction, self-assembly, hydrolysis behaviors

## Abstract

Studies on cerium oxo clusters (CeOCs) are not only significant for understanding the redox and hydrolysis behaviors of Ce(III/IV) ions but also crucial for the rational synthesis of novel clusters and nanoceria with specific Ce(III)/Ce(IV) ratios. Here, two sets of reactions were conducted using cerium nitrate and H_2_O_2_-oxidized cerium nitrate, resulting in the formation of two distinct mixed-valent CeOCs [Ce^III^_4_Ce^IV^_10_O_14_(OH)_2_(PhCO_2_)_22_(DMF)_6_] (Ce_14_) and [Ce^III^_2_Ce^IV^_22_O_28_(OH)_8_(PhCO_2_)_30_(DMF)_4_] (Ce_24C_). These two clusters exhibit different structures and Ce(III)/Ce(IV) ratios, demonstrating the critical role of cerium oxidation states and the occurrence of redox reactions in cluster formation. Ce_14_ is the first tetradecanuclear CeOC with a novel structure, whereas Ce_24C_ differed in its Ce(III)/Ce(IV) ratio, protonation levels of O atoms, and ligands from previously reported 24-nuclear CeOCs. Furthermore, various techniques were employed to investigate the formation process of these two clusters. X-ray photoelectron spectra (XPS) revealed that the white precipitates formed during the preparation of Ce_14_ contain Ce(III) ions, while the reddish-brown precipitates formed during the preparation of Ce_24C_ contain a mixture of Ce(III) and Ce(IV) ions. These two precipitations were individually dissolved in N,N-Dimethylformamide (DMF). The evolution of solution color and ultraviolet-visible (UV-Vis) spectra over time revealed the gradual oxidation of partial Ce(III) ions by oxygen in the solution of the white precipitation. As Ce(IV) ions increased in this solution, time-resolved small angle X-ray scattering (SAXS) data demonstrated the self-assembly of the Ce_14_ clusters after 4 days. In contrast, SAXS data and UV-Vis spectra revealed the rapid assembly of Ce_24C_ clusters within 2 h due to the initial coexistence of Ce(IV) and Ce(III) ions in the DMF solution of the reddish-brown precipitation. The continued reduction of partial Ce(IV) ions in this solution does not affect Ce_24C_ clusters’ formation and stability. Our studies expand the family of CeOCs and enhance our understanding of the effects of cerium’s oxidation states on cluster formation.

## 1 Introduction

Metal oxo clusters (MOCs) are polynuclear species that consist of several to hundreds of metal ions, bridged together by O^2-^, OH^−^, and/or H_2_O ligands (Nyman, 2016). MOCs have been extensively investigated owing to their diverse structures, intriguing properties, as well as broad applications in many fields (Qiu and Burns, 2013; Zheng et al., 2019; Long et al., 2010). One representative family of MOCs is lanthanide oxo clusters, which are typically formed due to the hydrolysis reaction of lanthanide ions (Zheng et al., 2019).

Cerium (Ce) is the most abundant lanthanide element (Chauvel, 2018). Unlike other lanthanide elements, cerium exhibits two stable oxidation states (+3 and +4), giving rise to its reversible redox behaviors observed in various compounds (Piro et al., 2014). This distinctive feature makes cerium materials have significant applications in diverse fields such as catalysis (Ahmad et al., 2020), energy (Fan et al., 2013), and biomedicine (Chen et al., 2024). Cerium ions readily undergo hydrolysis reactions to form cerium oxo clusters (CeOCs) (Hennig et al., 2013). CeOCs typically consist of cores Ce_x_ (O/OH)_y_ (where x is the count of cerium ions, and y is the count of O^2-^ and/or OH^−^ groups in the core), passivated by various organic/inorganic ligands including carboxylate (Hennig et al., 2013), phosphonate (Russell-Webster et al., 2021a), and sulfate (Colliard et al., 2021). These cores mostly exhibit a fluorite-like structure akin to cerium oxide, classifying CeOCs as a class of nanoceria with well-defined structures (Mitchell et al., 2017). Therefore, exploring CeOCs could not only advance our understanding of the hydrolysis behaviors of metal ions but also facilitate the rational design and synthesis of novel MOCs and nanoceria with controlled properties.

So far, CeOCs containing up to 100 cerium ions have been reported (Russell-Webster et al., 2021b). Compared to MOCs of transition metals and other lanthanides (Long and Cronin, 2021; Zheng et al., 2019), CeOCs have not been fully explored. It is worth mentioning that earlier research on CeOCs mainly focused on their syntheses and structures (Malaestean et al., 2012). Some recent studies have investigated the catalytic performance of specific CeOCs (Wasson et al., 2022). Nevertheless, studies investigating the assembly process of CeOCs remain limited. For example, Nyman’s group explored the roles of countercations in the self-assembly process of Ce_70_ and (Ce_62_)_2_ clusters (Colliard et al., 2021; Colliard et al., 2023) and the stacking mode of Ce_70_ clusters (Colliard and Nyman, 2021) using SAXS. Knope’s group investigated the transformation of a Ce_10_ cluster to a Ce_12_ cluster using electrospray ionization mass spectrometry and SAXS (Blanes-Díaz et al., 2024).

CeOCs are synthesized using various Ce(III) (Blanes-Díaz et al., 2024; Mathey et al., 2015) or Ce(IV) compounds (Mitchell et al., 2021; Mitchell et al., 2017) as reactants. Interestingly, these clusters consist of either solely Ce(IV) ions or a blend of Ce(III) and Ce(IV) ions in their structures, indicating the occurrence of *in-situ* redox reactions during the formation of certain clusters. However, *in-situ* redox reactions of cerium ions, especially the effects of these processes on the formation of CeOCs, remain largely unexplored, although such studies are significant for the understanding of the catalytic reactivity of CeOCs and the rational construction of novel nanoceria with tailored Ce(III)/Ce(IV) ratios. In addition, the variations in compositions of cerium compounds used as in previous studies might affect the formation and types of cerium clusters, thus posing a challenge in understanding the influence of cerium oxidation states on cluster formation.

In this study, we conducted two sets of reactions to investigate the effect of cerium oxidation states on cluster formation. To eliminate the effects of compositional differences from various cerium sources used in previous studies, we exclusively used cerium (III) nitrate. One set used it directly. In the other set, H_2_O_2_ was first employed to oxidize Ce(III) ions to Ce(IV) ions, as it does not introduce additional impurities (Djuričić and Pickering, 1999). The subsequent synthesis processes were identical. Ultimately, two distinct mixed-valent cerium oxo clusters, Ce_14_ and Ce_24C_, were formed from the two sets of reactions. Here, we report on their syntheses, structures, and formation processes.

## 2 Materials and methods

### 2.1 Materials

Cerium nitrate hexahydrate (Ce(NO_3_)_3_·6H_2_O, AR), anhydrous methanol (AR), ferric perchlorate (Fe(ClO_4_)_3_, AR), nitric acid (HNO_3_, 65–68 wt%, AR), hydrogen peroxide solution (H_2_O_2_, 30 wt%, AR), sodium benzoate (PhCOONa, AR), and dimethylformamide (DMF, AR) were purchased from Sinopharm Chemical Reagent Co., Ltd. and used as received. All solutions were prepared by using 18.25 MΩ deionized water.

### 2.2 Syntheses

#### 2.2.1 Synthesis of Ce_14_

Cerium nitrate hexahydrate (0.434 g, 1 mmol) was dissolved in anhydrous methanol (1 mL), resulting in a colorless solution. This solution was then mixed with a solution prepared by dissolving sodium benzoate (0.576 g, 4 mmol) in anhydrous methanol (20 mL). The resulting solution was stirred for 15 min, leading to the formation of a white precipitate (around 0.6 g), labeled as Precipitate-1. Precipitate-1 was filtered and added to DMF (10 mL), followed by stirring until fully dissolved. The resulting solution (pH ≈ 9.3) was left to evaporate under ambient conditions. After approximately 10 days, light yellow plate crystals (Supplementary Figure S1) containing Ce_14_ clusters were obtained.

The yield of crystals prepared using the above method was low. Numerous reactions were conducted to optimize the synthesis method. It was found that the addition of a ferric salt to the solution of cerium nitrate can increase the crystal yield. For example, ferric perchlorate (0.5 M, 150 μL) and nitric acid (0.5 M, 450 μL) were added to the solution that was prepared by dissolving cerium nitrate hexahydrate (0.434 g, 1 mmol) in anhydrous methanol (1 mL). The next steps were the same as above. After approximately 10 days, light yellow plate crystals containing Ce_14_ formed with a yield of 17.4% based on the cerium used.

#### 2.2.2 Synthesis of Ce_24C_

Cerium nitrate (0.434 g, 1 mmol) was dissolved in anhydrous methanol (1 mL). Subsequently, H_2_O_2_ (50 μL, 30 wt%) was added. The resulting reddish-brown solution was then mixed with a solution prepared by dissolving sodium benzoate (0.576 g, 4 mmol) in anhydrous methanol (20 mL). The resulting solution was stirred for 15 min, leading to the formation of a reddish-brown precipitate (around 0.58 g), labeled as Precipitate-2. Precipitate-2 was filtered and added to DMF (10 mL), followed by stirring until fully dissolved. The resulting solution (pH ≈ 8.6) was left to evaporate under ambient conditions. After approximately 8 days, light orange block crystals (Supplementary Figure S1) containing Ce_24C_ clusters formed with a yield of 18.1% based on the cerium used.

### 2.3 Instruments and characterization

#### 2.3.1 Single crystal X-ray diffraction measurements

Single crystal X-ray diffraction data for the crystals of Ce_14_ and Ce_24C_ were collected using a Bruker D8 Quest diffractometer with Mo Kα radiation (λ = 0.71073 Å). The structures were solved *via* the intrinsic phasing method with SHELXT (Sheldrick, 2015b) and refined using the SHELXL program (Sheldrick, 2015a). All non-hydrogen atoms could be located in the different Fourier maps and refined anisotropically. Types of O atoms, namely, O^2-^ and OH^−^ were assigned based on the bond valence sum (BVS) analysis. Most H atoms were positioned in idealized positions and refined as riding on their parent atoms and H atoms from μ_3_-OH in both Ce_14_ and Ce_24C_ were located using Fourier maps. H atoms from μ_4_-OH groups in Ce_24C_ could not be located using Fourier maps and were not included in the refinement of its structure. The disordered solvent molecules were removed by the SQUEEZE command. A total of 41 and 300 electrons were removed for the crystals of Ce_14_ and Ce_24C_, respectively. Elemental analysis suggested that these electrons are attributed to the presence of 5 and 7 DMF molecules. The crystallographic data were deposited at the Cambridge Crystallographic Data Centre with deposition codes CCDC 2328826 and 2328827 for Ce_14_ and Ce_24C_, respectively.

#### 2.3.2 Composition and property measurements of crystals and precipitations

The powder X-ray diffraction (PXRD) data for the crystals of Ce_14_ and Ce_24C_, as well as the precipitations formed during their preparation process, were collected using a Bruker D8 Advance diffractometer with Cu Kα radiation (λ = 1.54056 Å), at a scan rate of 0.05°/s. Additionally, XPS data of these crystals and precipitations were measured using a Thermo Fisher ESCALAB Xi + spectrometer equipped with a monochromatic Aluminum 400 W X-ray source.

The contents of Ce in the crystals of Ce_14_ and Ce_24C_ were measured using a PerkinElmer NexION 350D inductively coupled plasma mass spectrometer (ICP-MS). The contents of C, H, and N in these crystals were measured using a EUROVECTOR EA3000 elemental analyzer. Anal. calcd (found) for crystals of Ce_14_ (C_199_H_217_Ce_14_N_15_O_75_): Ce, 32.79% (33.20%); C, 39.97% (39.66%); H, 3.66% (3.45%); N, 3.51% (3.96%). Anal. Calcd (found) for crystals of Ce_24C_ (C_282_H_326_Ce_24_N_24_O_120_): Ce, 36.03% (36.09%); C, 36.29% (36.36%); H, 3.52% (3.95%); N, 3.60% (3.91%).

Fourier transform infrared (FTIR) spectra of the crystals of Ce_14_ and Ce_24C_ were recorded using a Bruker VERTEX 70 spectrometer. Their Raman spectra were acquired using a Renishaw inVia Qontor spectrometer with an excitation wavelength of 532 nm. Their thermogravimetric analysis (TGA) data were collected by heating crystals (Ce_14_: 2.33 mg, Ce_24C_: 2.89 mg) from 35°C to 1,200°C at a rate of 10 °C/min under flowing N_2_ in a Mettler Toledo TGA/DSC3+ instrument.

#### 2.3.3 Measurements of reaction solutions

Time-resolved UV-Vis spectra of the DMF solutions of Precipitate-1 and Precipitate-2 with reaction times from 0 h (immediately after the completion of the reaction) to 8 days were recorded using a GENESYS 150 spectrophotometer. Time-resolved SAXS data of these solutions were collected using an Anton Paar SAXSpoint 2.0 instrument with Cu Kα radiation. The solutions were individually placed into glass capillaries and then put on the sample stage. The distance between the sample stage and the detector was 250 mm. The exposure time was 30 min. SAXS data of pure DMF solution was also collected under the same condition for the background subtraction.

## 3 Result and discussion

### 3.1 Syntheses of Ce_14_ and Ce_24C_

To eliminate the effects of compositional differences from different cerium sources, all synthesis reactions were conducted exclusively using cerium nitrate. To change the oxidation states of cerium ions, the H_2_O_2_ solution was employed for oxidizing cerium nitrate. We found that the Ce_14_ crystals are formed from reactions using cerium nitrate. In contrast, the Ce_24C_ crystals are formed from reactions using cerium nitrate that was oxidized by the H_2_O_2_ solution. These observations suggest that the oxidation states of cerium ions in reactants play an important role in the formation and types of CeOCs.

The initial yield of Ce_14_ crystals was low. Therefore, we conducted numerous reactions to optimize the synthesis method and found that the addition of appropriate ferric salts and nitric acid could be beneficial. Their addition increases the acidity of the reaction solution and may affect the oxidation process of Ce(III) ions. However, the exact function of the ferric salts and nitric acid remains uncertain, despite our efforts to understand it through various studies. It is noteworthy that Fe(NO_3_)_3_ has been previously used in syntheses of CeOCs (Mitchell et al., 2021).

### 3.2 Structures of Ce_14_ and Ce_24C_

**FIGURE 1:**
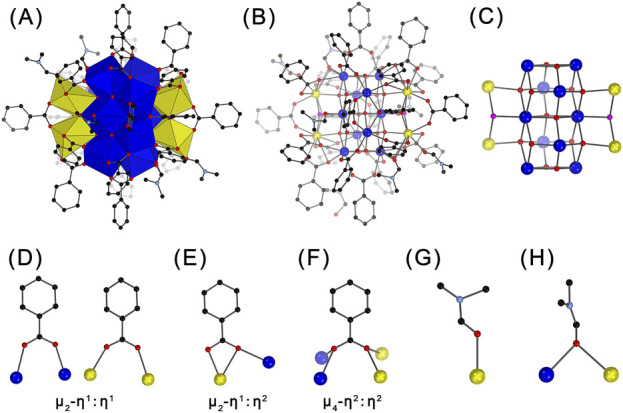
Polyhedral **(A)** and ball-and-stick **(B–H)** representations of the structure of Ce_14_ **(A, B)**, its core **(C)**, the coordination modes of benzoate groups **(D–F)**, and the coordination modes of DMF molecules **(G–H)**. Ce(IV) ions are shown in blue; Ce(III) ions in yellow, O^2-^ ions in red; OH^−^ ions in pink; C atoms in black; N atoms in light blue. H atoms are omitted for clarity.

**FIGURE 2:**
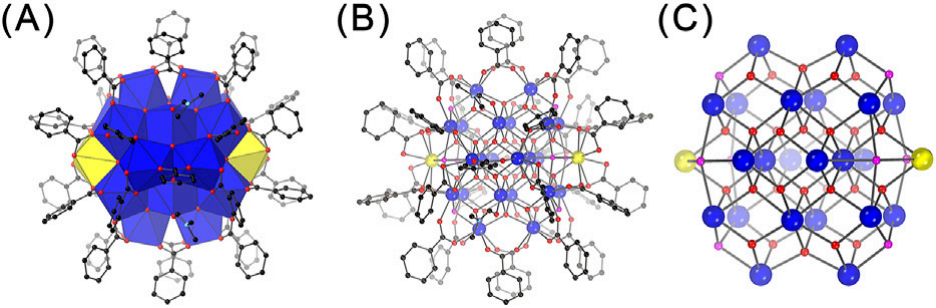
Polyhedral **(A)** and ball-and-stick **(B, C)** representations of the structure of Ce_24C_ **(A, B)** and its core **(C)**. Legends as that in Figure 1.

The crystallographic analysis revealed that crystals of Ce_14_ and Ce_24C_ containing two distinct types of CeOCs with different structures and Ce(III)/Ce(IV) ratios (Figures 1A, 2A). Specifically, Ce_14_ cluster crystallizes in the triclinic space group *P*-1 (Supplementary Table S1). Its structure comprises 14 cerium ions, with four of them being Ce(III) ions and the remaining ten being Ce(IV) ions, as demonstrated by BVS calculations (Supplementary Table S2). To our knowledge, Ce_14_ is the first tetradecanuclear CeOC. All ten Ce(IV) ions are eight-coordinated, forming Ce-O bonds with distances ranging from 2.157 Å to 2.586 Å (Supplementary Table S4). In contrast, two Ce(III) ions are nine-coordinated and the other two are ten-coordinated, with much longer Ce-O bond distances spanning from 2.387 Å to 2.968 Å. As shown in Figure 1B, the ten Ce(IV) ions are connected through bridging O^2-^ groups, forming a decamer. The four Ce(III) ions are divided into two groups and situated on opposite sides of the decamer through connections of μ_4_-O^2-^ and μ_3_-OH^-^ groups, resulting in the formation of Ce_14_ core (Figures 1B, C; Supplementary Table S3). Similar to cores of other CeOCs, this core also exhibits a fluorite structure (Supplementary Figure S2).

The core of Ce_14_ is coordinated by 22 PhCO_2_^-^ and six DMF groups, forming the complete structure of the Ce_14_ cluster with a formula of [Ce^III^_4_Ce^IV^_10_O_14_(OH)_2_(PhCO_2_)_22_(DMF)_6_]. The coordination modes of PhCO_2_^-^ groups are quite diverse. Twelve of them adopt the common μ_2_-η^1^:η^1^-bridging mode (Figure 1D), eight exhibit the μ_2_-η^1^:η^2^ chelating and bridging mode (Figure 1E), and the remaining two adopt a rare μ_4_-η^2^:η^2^-bridging mode (Figure 1F) which was only observed in cluster Ce_100_ (Russell-Webster et al., 2021b). The six DMF groups exhibit two coordination environments. Four of them bond to one Ce ion through the O atom, while the remaining two bridge two adjacent Ce ions through the O atom (Figures 1G,H). The crystallographic analysis also revealed the presence of DMF molecules dispersed among the Ce_14_ clusters (Supplementary Figure S3). The elemental analysis indicated approximately 9 DMF molecules per cluster. Thus, the Ce_14_ crystal has a formula of [Ce^III^_4_Ce^IV^_10_O_14_(OH)_2_(PhCO_2_)_22_(DMF)_6_]·9DMF.

The combination of the crystallographic and elemental analyses revealed that Ce_24C_ is a 24-nuclear cluster containing two Ce(III) ions and 22 Ce(IV) ions, and it crystallizes with DMF molecule in the *P*-1 space group to form crystals with a formula of [Ce^III^_2_Ce^IV^_22_O_28_(OH)_8_(PhCO_2_)_30_(DMF)_4_]·20DMF (Figure 2; Supplementary Figure S4; Supplementary Table S1; Supplementary Tables S5-S7). The structure of Ce_24C_ is similar to those of previously reported Ce_24A_ (Mitchell et al., 2017) and Ce_24B_ (Mitchell et al., 2021) clusters. The primary differences among these three clusters lie in the ratio of Ce(III)/Ce(IV) ions, the number of OH^−^ groups, and the type of ligands on their surface. The ratio of Ce(III)/Ce(IV) ions in the cores of Ce_24A_ and Ce_24C_ is 2:22, while that in Ce_24B_ is 3:21. Both Ce_24A_ and Ce_24C_ contain eight OH^−^ groups, while Ce_24B_ contains nine OH^−^ groups. The core of Ce_24C_ is capped by 30 PhCO_2_^-^ and four DMF groups, whereas Ce_24A_ and Ce_24B_ feature four pyridine groups instead of DMF groups. These structural variations demonstrate that the protonation levels of O atoms and the amount of Ce(III) ions in the cores of cerium oxo clusters are variable, and their surface ligands are replaceable. Similar results have been observed in other metal oxo clusters (Zhang et al., 2021).

### 3.3 Characterizations of Ce_14_ and Ce_24C_ crystals

**FIGURE 3:**
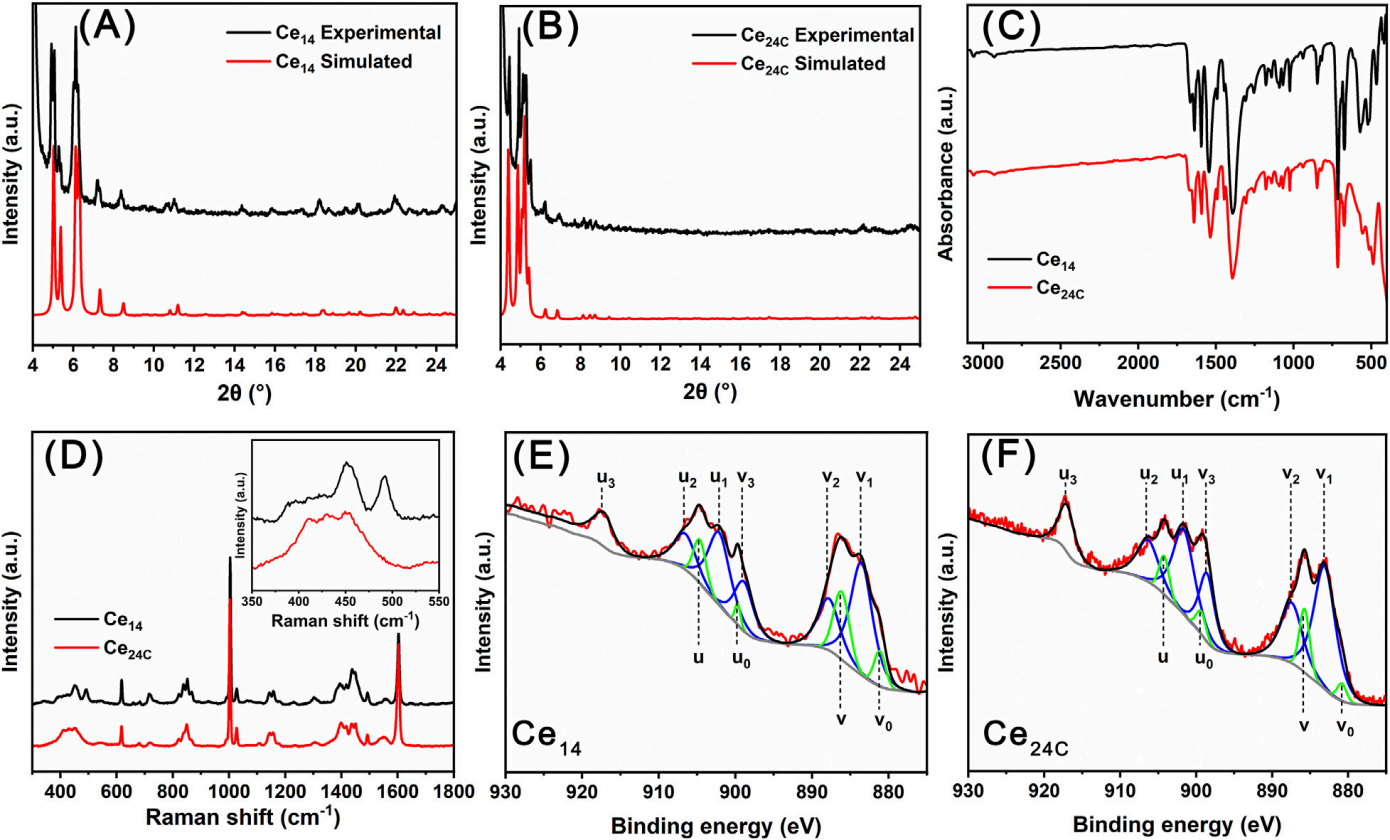
PXRD patterns **(A, B)**, FTIR spectra **(C)**, Raman spectra **(D)**, and XPS data **(E, F)** for Ce_14_ and Ce_24C_ crystals.

Crystals of Ce_14_ and Ce_24C_ were characterized using PXRD, FTIR, TGA, Raman spectroscopy, and XPS to confirm their chemical composition and oxidation states of cerium ions within their structures. Specifically, the experimental PXRD patterns match well with simulated patterns calculated using single crystal X-ray diffraction data for both Ce_14_ and Ce_24C_, indicating that crystals of both Ce_14_ and Ce_24C_ are pure (Figures 3A,B). FTIR spectra for crystals of Ce_14_ and Ce_24C_ are similar (Figure 3C). Assignments of main IR peaks were provided in Supplementary Table S8. These peaks demonstrated the presence of Ce-O bonds, benzoate groups, and DMF molecules within these crystals. Their Raman spectra also exhibit the same features (Figure 3D and the inset). The peak at 451 cm^-1^ is related to the F_2g_ mode of the Ce-O-Ce bond, which is formed due to the hydrolysis and condensation reactions of cerium ions (Miller and Irish, 1967; Weber et al., 1993). The peaks at 618 and 718 cm^-1^ are related to the out-of-plane deformation of the C=O and C-H bonds in the benzoate group, respectively (Klausberger et al., 1977). The peak at 660 cm^-1^ is attributed to the bending vibration of the O=C-N group in the DMF molecule (Kislina et al., 1994). The peak at 680 cm^-1^ corresponds to the in-plane deformation of the aromatic ring in the benzoate group (Klausberger et al., 1977). The peaks located at 1,003 and 1,604 cm^-1^ are related to the vibration of the phenyl ring of the benzoate group, and those at 1,418 and 1,550 cm^-1^ are related to the symmetric and asymmetric vibration of CO_2_^−^ groups of the benzoate group (Lewandowski and Baranska, 1986). Moreover, as observed in their XPS spectra (Figures 3E,F), the 3d_5/2_ and 3d_3/2_ peaks of cerium are located within the ranges of 877–890 eV and 897–910 eV, respectively. The spin-orbit splitting of these two peaks, along with the satellite peak located at 917 eV, confirms the coexistence of Ce(III) and Ce(IV) ions in the structures of Ce_14_ and Ce_24C_ (Wacker et al., 2022). Specifically, in the spectrum of Ce_14_, the peaks at 881.2 (v_0_), 886.2 (v), 899.7 (u_0_), and 904.6 (u) eV are ascribed to Ce(III) ions, while the peaks at 883.6 (v_1_), 887.8 (v_2_), 898.9 (v_3_), 902.1 (u_1_), 906.6 (u_2_), and 917.2 (u_3_) eV are ascribed to Ce(IV) ions (Wacker et al., 2022). In the spectrum of Ce_24C_, the peaks at 880.8 (v_0_), 885.7 (v), 899.4 (u_0_), and 904.2 (u) eV are ascribed to Ce(III) ions, while the peaks at 883.1 (v_1_), 887.4 (v_2_), 898.6 (v_3_), 901.7 (u_1_), 906.3 (u_2_), and 917.0 (u_3_) eV are ascribed to Ce(IV) ions (Wacker et al., 2022).

### 3.4 Self-assembly of Ce_14_ and Ce_24C_ clusters

As mentioned above, both Ce_14_ and Ce_24C_ clusters exhibit mixed valence, even though they were synthesized using Ce(NO_3_)_3_ and oxidized Ce(NO_3_)_3_, respectively. This suggests that cerium ions undergo redox reactions during the preparation process of these clusters. Therefore, we investigated the effects of redox reactions of cerium ions on the formation process of Ce_14_ and Ce_24C_ using various techniques.

**FIGURE 4:**
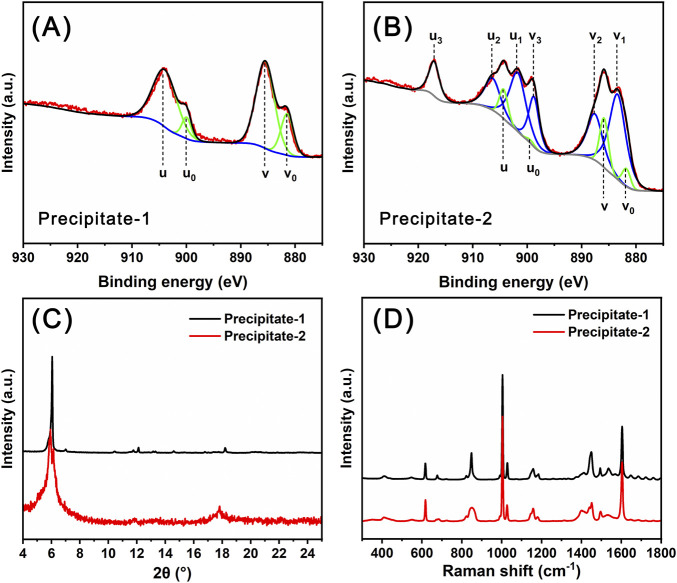
XPS data **(A, B)**, PXRD patterns **(C)**, and Raman spectra **(D)** for Precipitate-1 and Precipitate-2.

In the preparation process of Ce_14_, the combination of solutions of Ce(NO_3_)_3_ and sodium benzoate yielded a white precipitate, designated as Precipitate-1. In contrast, during the preparation of Ce_24C_, the combination of solutions of Ce(NO_3_)_3_, H_2_O_2_, and sodium benzoate yielded a reddish-brown precipitate, designated as Precipitate-2. In the XPS spectrum of Precipitate-1 (Figure 4A), all four peaks at 881.8 (v_0_), 885.5 (v), 900.0 (u_0_), and 904.2 (u) eV are ascribed to Ce(III) ions (Wacker et al., 2022). In contrast, in the XPS spectrum of Precipitate-2 (Figure 4B), the peaks at 881.8 (v_0_), 885.9 (v), 899.4 (u_0_), and 904.3 (u) eV are ascribed to Ce(III) ions, while the peaks at 883.4 (v_1_), 887.6 (v_2_), 898.8 (v_3_), 901.7 (u_1_), 906.4 (u_2_), and 917.1 (u_3_) eV are ascribed to Ce(IV) ions (Wacker et al., 2022). These XPS spectra (Figure 4A) demonstrate the presence of Ce(III) ions in Precipitate-1 and the coexistence of Ce(IV) ions along with a small amount of Ce(III) ions in Precipitate-2 (Figure 4B). One possible reason for this coexistence is the incomplete oxidation of Ce(III) ions by H_2_O_2_. Another reason could be that the Ce(IV) ions generated through the oxidation of Ce(III) ions by H_2_O_2_ are unstable and prone to partially transforming back to Ce(III) ions (Das et al., 2007; Lee et al., 2013). Both PXRD patterns and Raman spectra (Figures 4C,D) of Precipitate-1 and Precipitate-2 differ from those of Ce_14_ and Ce_24C_ crystals. Additionally, the Raman spectra indicate the incorporation of benzoate groups into the precipitates (Lewandowski and Baranska, 1986). These findings suggest that Precipitate-1 and Precipitate-2 are intermediate cerium benzoate complexes formed during the synthesis of Ce_14_ and Ce_24C_, respectively.

**FIGURE 5:**
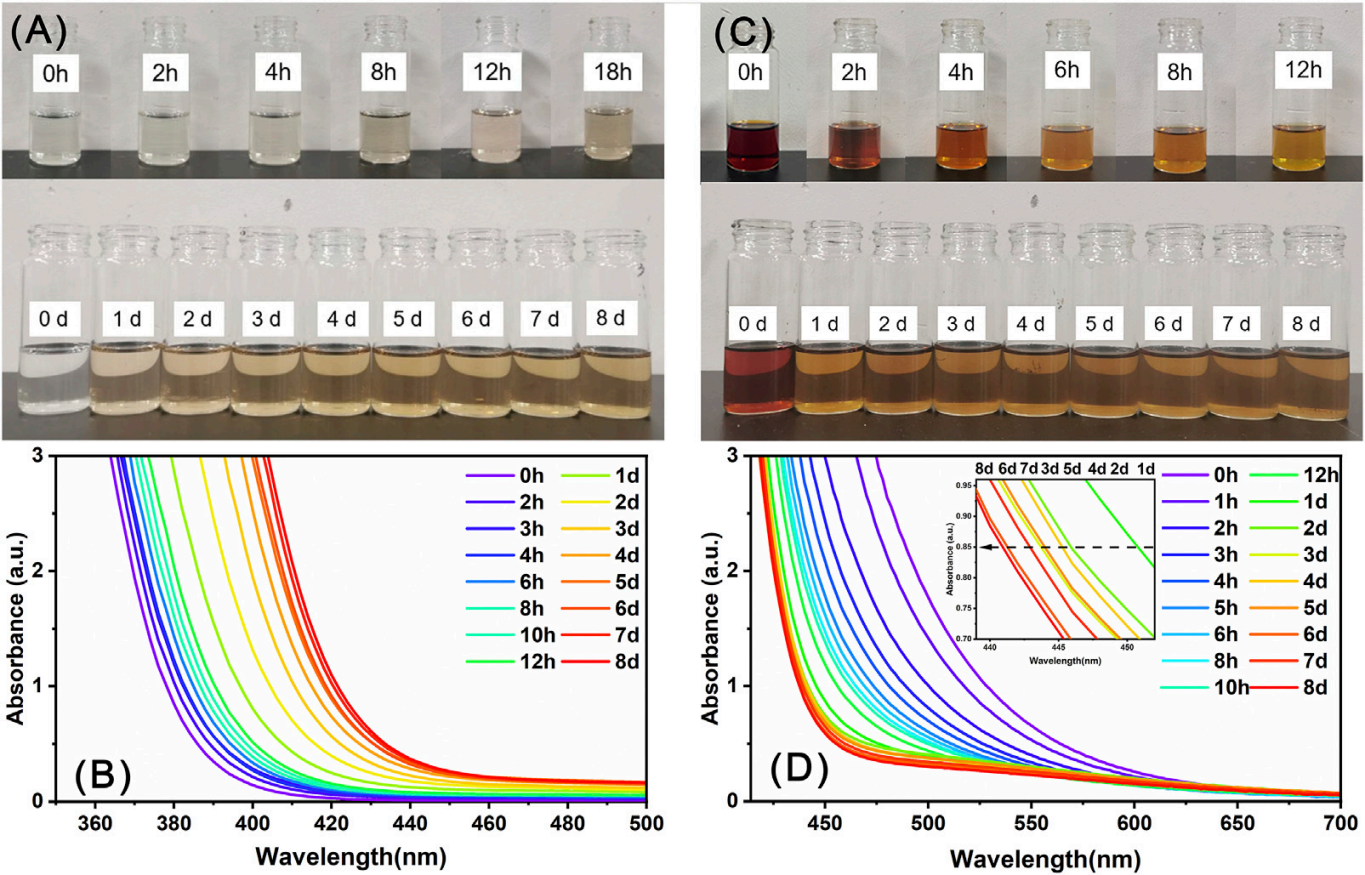
The evolution of the color **(A, C)** and UV-Vis spectra **(B, D)** of the DMF solutions of Precipitate-1 **(A, B)** and Precipitate-2 **(C, D)** over time.

Precipitate-1 and Precipitate-2 were individually dissolved in DMF, yielding a colorless and reddish-brown solution, respectively. These two solutions were subsequently allowed to evaporate under ambient conditions. As shown in Figure 5A, the solution color of Precipitate-1 gradually changed from colorless to brownish-yellow over time and essentially no longer changed after the solution was stood for about 4 days. The corresponding time-resolved UV-Vis spectra (Figure 5B) exhibit a pronounced red shift in the spectra over time, particularly during the initial 4 days. These observations indicate the gradual oxidation of Ce(III) ions to Ce(IV) ions in the DMF solution of Precipitate-1 (Das et al., 2007; Lee et al., 2013). Considering the absence of strong oxidizing agents in this solution, the oxidation of Ce^3+^ ions likely occurs due to exposure to atmospheric oxygen. To eliminate the presence of oxygen, the solution was placed in a nitrogen-filled glove box. After 15 days, the solution remained colorless, and no crystals were observed. This outcome strongly suggests that the presence of molecular oxygen is crucial for the oxidation of Ce(III) ions.

In contrast, the solution color of Precipitate-2 lightened at a faster rate and nearly stabilized after about 1 day (Figure 5C). The corresponding time-resolved UV-Vis spectra (Figure 5D) exhibited a noticeable blue shift within 1 day, suggesting that partial Ce(IV) ions in the solution are rapidly reduced to Ce(III) ions (Das et al., 2007; Perez et al., 2008). After a day, a noticeable oscillation appears in the absorption spectrum around 445 nm (The insert in Figure 5D), indicating that the reduction of Ce(IV) ions and oxidation of Ce(III) ions occurred simultaneously in the solution and the Ce(III)/Ce(IV) ratio eventually reached dynamic equilibrium (Perez et al., 2008). Notably, the final stable color of the DMF solution of Precipitate-1 was lighter than that of Precipitate-2, indicating a higher Ce(III/IV) ratio in the former (Perez et al., 2008). This is probably why the Ce(III/IV) ratio in Ce_14_ is higher than that in Ce_24C_. Furthermore, the faster stabilization of the color and UV-Vis spectra for the DMF solution of Precipitate-2 suggests that the reduction reaction of Ce(IV) ions in this solution occurred at a faster rate.

Generally, the Ce(III) state is favored under acidic conditions with poor donor ligands, while the Ce(IV) state is favored under basic conditions with strong donor ligands, particularly anionic oxygen donor ligands (Piro et al., 2014). Ce(NO_3_)_3_ is an acidic chemical, whereas sodium benzoate is weakly basic (Rived et al., 1998). Mixing methanol solutions of these two compounds causes Ce(III) ions to react with benzoate groups, forming Precipitate-1 in a relatively basic environment compared to the acidity of Ce(NO_3_)_3_ solid. DMF is considered a basic solvent due to its strong hydrogen-bond-accepting property (Kütt et al., 2018). Eventually, the dissolution of Precipitate-1 in DMF results in a weakly basic solution with a pH of 9.3. This indicates that Ce(III) ions in the DMF solution of Precipitate-1 are in a more basic condition compared to their initial state in Ce(NO_3_)_3_ solid. Furthermore, the benzoate group is a strong anionic oxygen donor ligand for cerium ions, as evidenced by our Raman spectra. Therefore, the oxidation of Ce(III) ions in the DMF solution of Precipitate-1 is likely driven by the basic solution environment and their complexation with benzoate groups. Notably, previously reported synthesis reactions of mixed-valent CeOCs using cerium (III) salts also employed neutral or alkaline solvents like methanol, acetonitrile, and pyridine, along with anionic oxygen donor ligands such as benzoate (Mitchell et al., 2021), acetylacetone (Blanes-Díaz et al., 2024), pivalate (Mathey et al., 2015), and isobutyrate (Malaestean et al., 2012; Canaj et al., 2017) groups. The exact reduction mechanism of Ce(IV) ions during CeOCs synthesis remains unclear. Blanes-Díaz et al. proposed that the reduction of Ce(SO_4_)_2_ during the formation of Ce_10_ may occur through the oxidation of acetylacetone (Blanes-Díaz et al., 2024). However, they did not provide conclusive evidence. Here, we propose the reduction of partial Ce^4+^ ions by DMF in this solution. DMF has been shown to act as a reducing agent in reduction reactions of certain transition metal ions, for example, transforming Ag^+^ ions to Ag^0^ and Cu^2+^ to Cu^+^ ions (Muzart, 2009).

**FIGURE 6:**
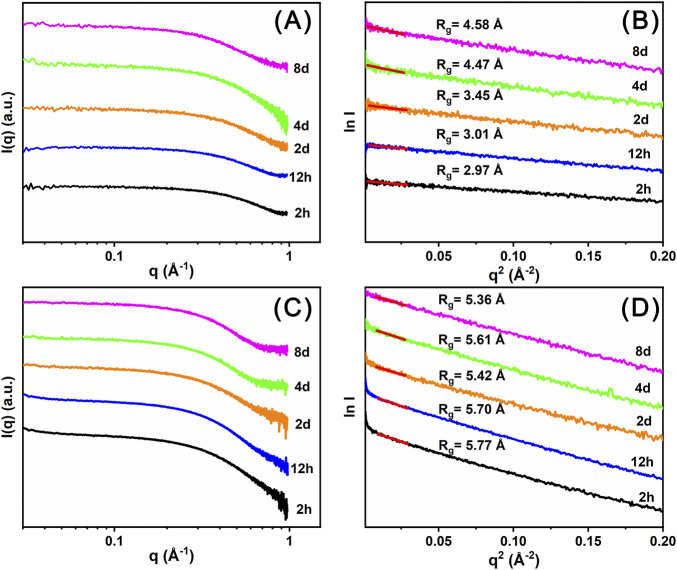
Time resolved SAXS plots **(A, C)** and corresponding Guinier analysis plots **(B, D)** of the DMF solutions of Precipitate-1 **(A, B)** and Precipitate-2 **(C, D)**.

Time-resolved SAXS data (Figures 6A,C) were collected to investigate the self-assembly process of Ce_14_ and Ce_24C_ clusters in the DMF solutions. Based on these experimental data, R_g_ values were calculated using Guinier analysis (Feigin et al., 1987). As shown in Figure 6B, the R_g_ value for the 2 h solution of Precipitate-1 is 2.97 Å, which is smaller than that (4.94 Å) of the Ce_14_ core, as calculated using single crystal diffraction data and the CRYSOL method (Svergun et al., 1995). This observation indicates that Ce_14_ has not formed at 2 h. According to the solution color and UV-Vis spectra introduced above, the insufficient oxidation of Ce^3+^ ions and low concentration of Ce(IV) ions in the solution at this time may hinder the formation the Ce_14_. By day 4, the R_g_ value increases to ∼4.47 Å which is closer to that of the Ce_14_ core, indicating that Ce_14_ clusters had begun to form. As indicated by the above-mentioned solution color and UV-Vis spectra, the ratio of Ce(III)/Ce(IV) nearly reaches dynamic equilibrium at day 4, which may facilitate the formation of Ce_14_ clusters. This conclusion is further supported by the R_g_ value (∼4.58 Å) for the day 8 solution. In contrast, R_g_ values for different time-aged (from 2 h to 8 days) solutions of Precipitate-2 are almost the same and around 5.61 Å (Figure 6D), which is comparable with that (5.35 Å) of the Ce_24C_ core calculated using the CRYSOL method. This finding suggests that the Ce_24C_ cluster rapidly assembles within 2 h and keeps stable in the solution after its formation. As demonstrated by the XPS data, Ce(IV) ions coexist with a small amount of Ce(III) ions in Precipitate-2. This coexistence may facilitate the rapid formation of Ce_24C_. Although some Ce(IV) ions are continuously reduced over several days, this reduction process does not hinder the formation and stability of Ce_24C_.

The formation of CeOC requires the hydrolysis of cerium ions to form a Ce_x_ (O/OH)_y_ core (Hennig et al., 2013). CeOCs in this study and previously reported studies are composed either entirely of Ce(IV) ions or of a mixture of Ce(IV) ions and a small number of Ce(III) ions, indicating that the formation of CeOCs mainly relies on the hydrolysis reactions of Ce(IV) ions due to their stronger hydrolysis ability compared to Ce(III) ions. After the dissolution of Precipitate-1 in DMF, the solution only contains Ce(III) ions and the transformation of Ce(III) ions to Ce(IV) ions takes time. Thus the formation of Ce_14_ requires several days. In contrast, after the dissolution of Precipitate-2 in DMF, the solution contains a high concentration of Ce(IV) ions and a small amount of Ce(III) ions, which is why Ce_24C_ can self-assemble rapidly. Additionally, this study may indicate multiple roles of DMF in the formation of the two cerium clusters, including acting as surface ligands to stabilize the clusters, providing a basic environment to facilitate the oxidation of Ce(III) ions, and reducing some Ce(IV) ions to maintain a dynamic equilibrium in the Ce(III)/Ce(IV) ratio.

## 4 Conclusion

In summary, two sets of reactions were conducted using cerium nitrate and H_2_O_2_-oxidized cerium nitrate, resulting in the formation of two distinct mixed-valent cerium oxo clusters, Ce_14_ and Ce_24C_. Ce_14_ represents the first tetradecanuclear cerium oxo cluster with a unique structure. Ce_24C_ is structurally similar to the two previously reported 24-nuclear cerium clusters. The structural difference among these three 24-nuclear clusters demonstrates that the protonation levels of O atoms and the Ce(III)/Ce(IV) ratio in the cores of cerium clusters are variable, and their surface ligands are replaceable. Furthermore, the formation process of Ce_14_ and Ce_24C_ clusters was investigated using various techniques. The PXRD and Raman data indicated that the white and reddish-brown precipitations formed during their preparation process are intermediate cerium benzoate complexes. The XPS data proved that these two precipitations contain Ce(III) ions and a mixture of Ce(III) and Ce(IV) ions, respectively. Time-resolved UV-Vis spectra demonstrated the gradual oxidation of partial Ce(III) ions by oxygen in the DMF solution of the white precipitation. With the continued increase of Ce(IV) ions, Time-resolved SAXS data indicated that the Ce_14_ clusters begin to form in the solution at day 4. In contrast, the initial coexistence of Ce(IV) ions and a small amount of Ce(III) ions in the DMF solution of the reddish-brown precipitation facilitate the rapid formation of Ce_24C_ clusters within 2 h.

Similar to nanoceria, both Ce_14_ and Ce_24C_ exhibit a fluorite-type structure containing Ce^4+^ and Ce^3+^ ions, suggesting their potential catalytic activity. Ongoing investigations are underway to explore their catalytic capabilities.

## Data Availability

The datasets presented in this study can be found in online repositories. The name of the repository and accession number(s) can be found in the article and Supplementary Material.
