# Supplementary material for: Two mixed-valent cerium oxo clusters: synthesis, structure, and self-assembly
Source: Front Chem. 2024 Dec 2;12:1507834. doi: 10.3389/fchem.2024.1507834 (PMC11646716; doi:10.3389/fchem.2024.1507834)
Supplement: Supplementary file 1 [file DataSheet1.pdf]

Supplementary Materials

Two Mixed-Valent Cerium Oxo Clusters: Synthesis, Structure, and Self-assembly

Yuan Gao[1], Yang Zhang[1], Zhe Han[1], Chunhui Wang[1], Lei Zhang[2], Jie Qiu[1*]

[1]School of Energy and Power Engineering, Xi'an Jiaotong University, Xi'an 710049, China.

[2] Engineering Laboratory of Advanced Energy Materials, Ningbo Institute of Materials Technology and Engineering, Chinese Academy of Sciences, Ningbo 315201, China.

1. Supplementary Figures

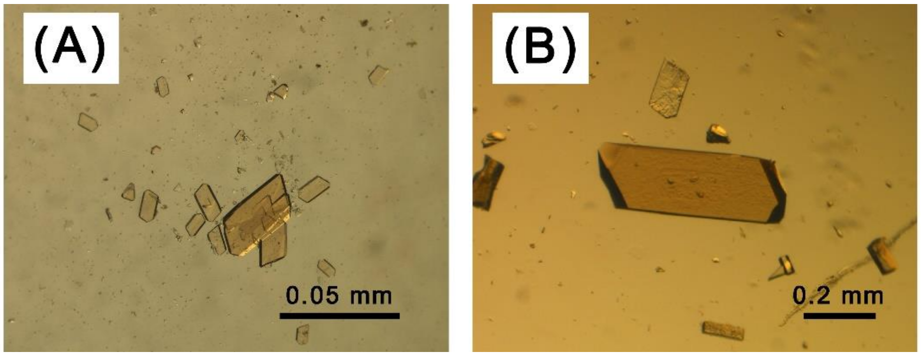

Supplementary Figure 1. Crystals containing Ce_{14} (a) or Ce_{24C} (b) clusters in their mother solution. The scale bar is 0.2 mm.

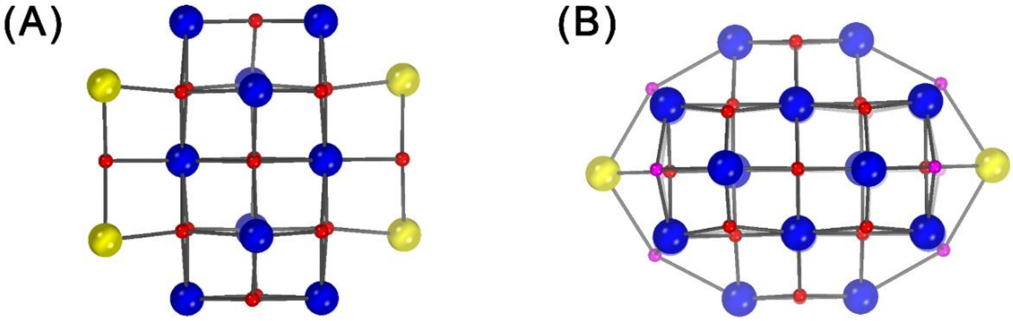

Supplementary Figure 2. The fluorite-type arrangement of cerium and oxygen atoms in the core of Ce_{14} and Ce_{24C}. Ce^{3+} ions, yellow; Ce^{4+} ions, blue; O atoms of OH^- groups, pink; other O atoms, red.

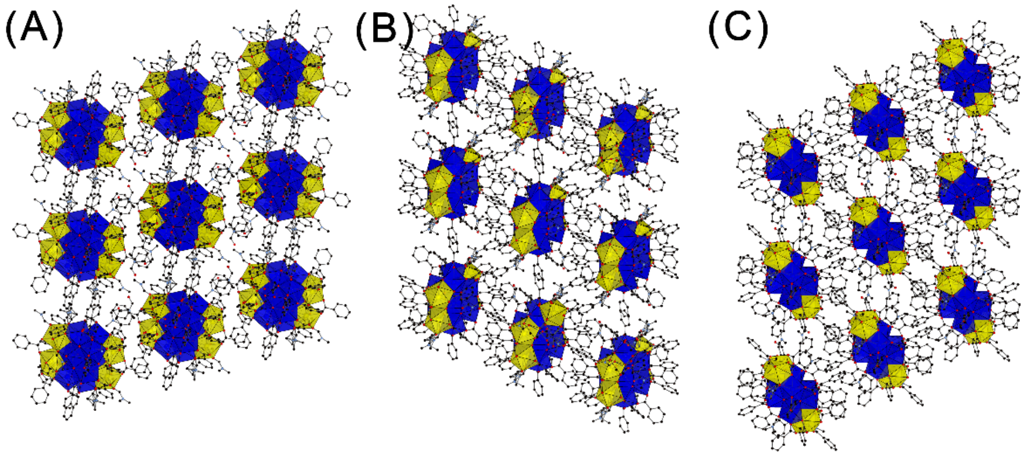

Supplementary Figure 3. The arrangement of Ce_{14} clusters viewed along the X (A), Y (B), and Z (C) axes respectively. Ce^{3+} ions, yellow; Ce^{4+} ions, blue; C atoms, black; O atoms of OH^- groups, pink; other O atoms, red; N atoms, light blue.

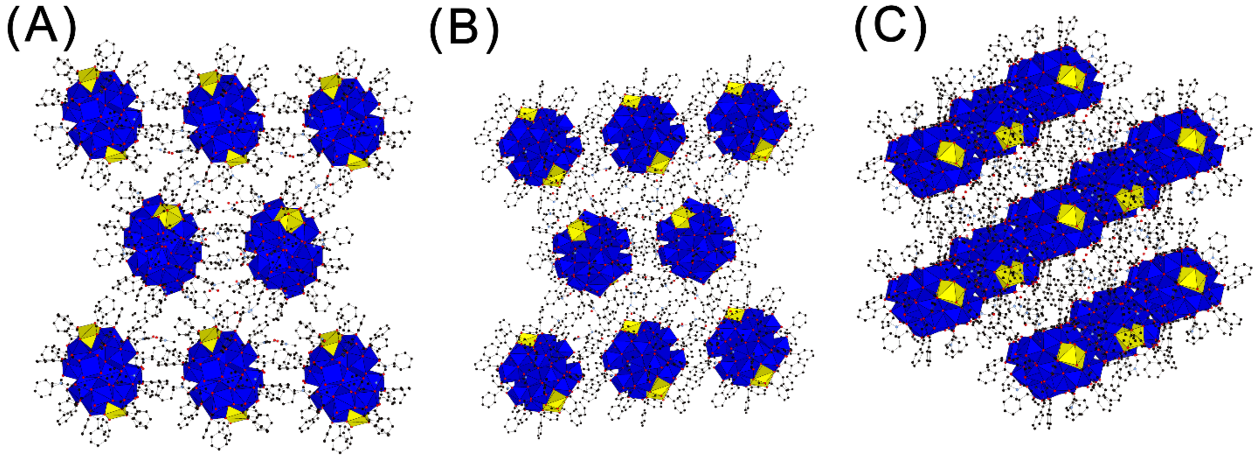

Supplementary Figure 4. The arrangement of Ce_{24C} clusters viewed along the X (A), Y (B), and Z (C) axes, respectively. Legend as in Supplementary Figure 3.

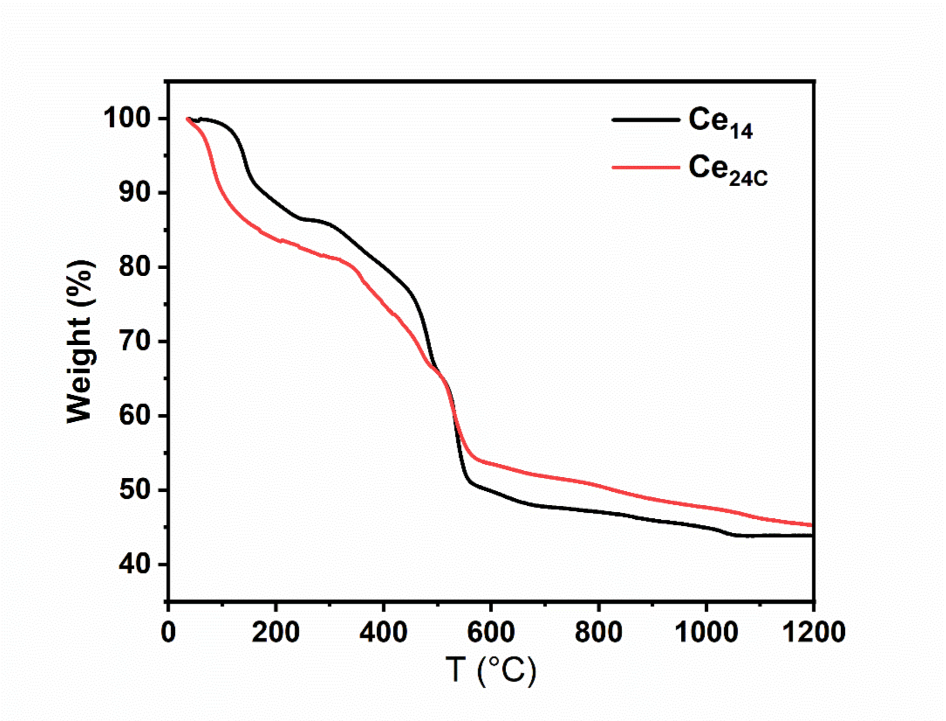

Supplementary Figure 5. The TGA thermograms of crystals containing clusters Ce_{14} and Ce_{24C}.

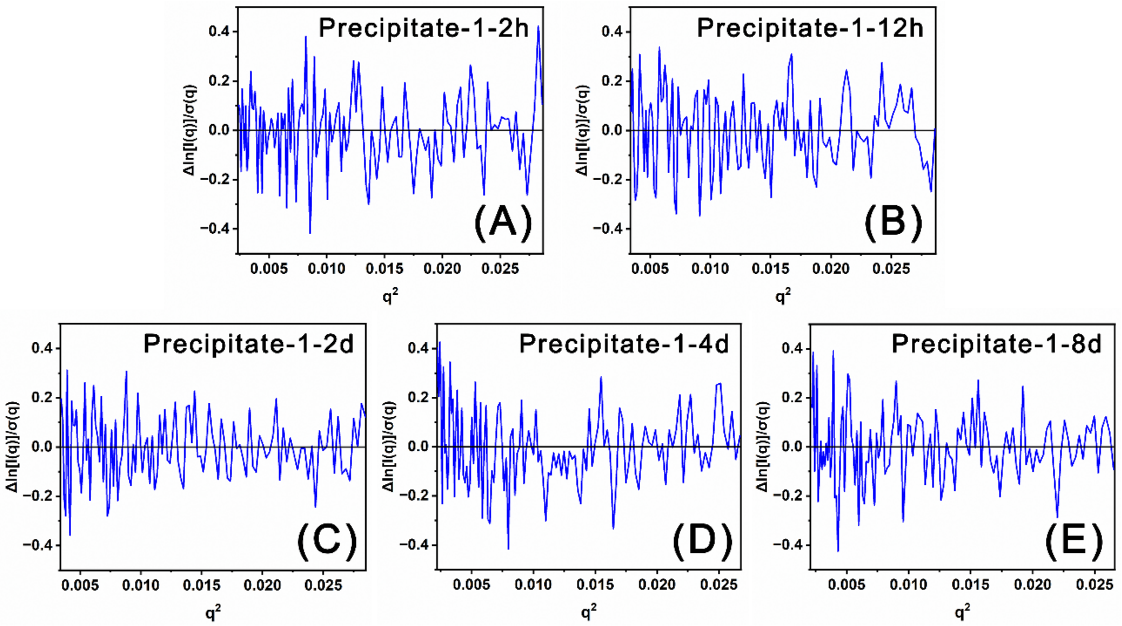

Supplementary Figure 6. Residuals from the Guinier analysis of SAXS data for the DMF solution of Precipitate-1.

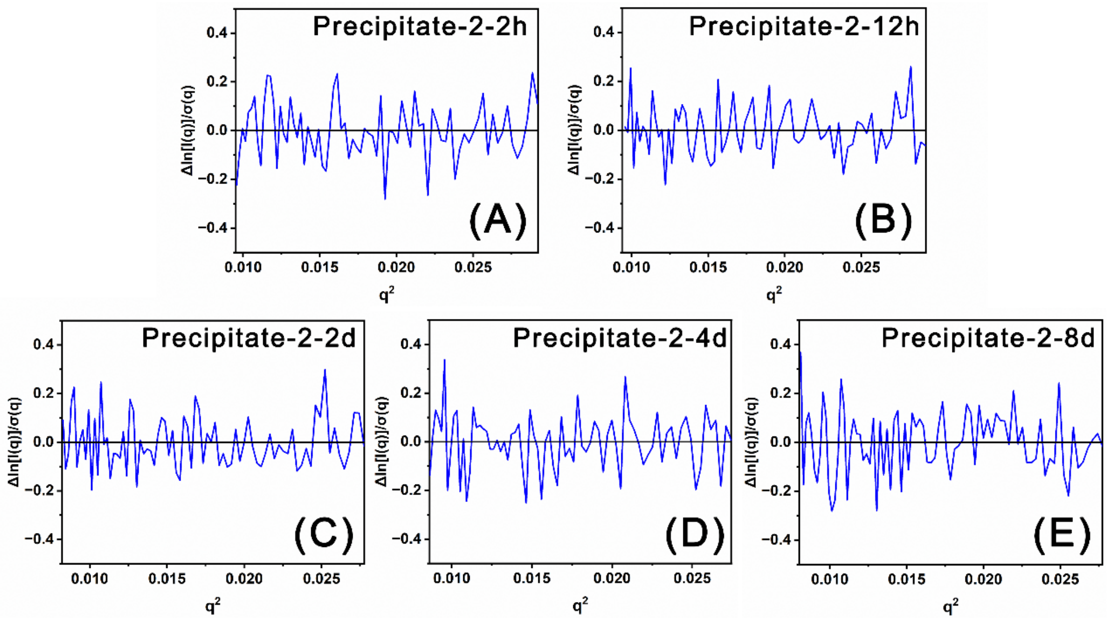

Supplementary Figure 7. Residuals from the Guinier analysis of SAXS data for the DMF solution of Precipitate-2.

2. Supplementary Tables

Supplementary Table 1. Crystallographic details for Ce_{14} and Ce_{24C} crystals.

Compound	Ce_{14}	Ce_{24C}
Formula	$C_{184}H_{182}Ce_{14}N_{10}O_{70}$	$C_{261}H_{273}Ce_{24}N_{17}O_{113}$
Formula weight	5615.07	8818.83
Crystal system	Triclinic	Triclinic
Space group	P-1	P-1
a [Å]	16.6470(9)	20.8903(11)
b [Å]	18.6577(9)	21.1877(11)
c [Å]	19.2054(11)	39.997(2)
α [°]	97.823(2)	84.403(2)
β[°]	107.677(2)	81.216(2)
γ[°]	112.987(2)	62.043(1)
V [Å^3]	5006.7(5)	15447.0(15)
Z	1	2
D (calc) [g/cm^3]	1.862	1.896
μ (Mo Kα) [/mm]	3.198	3.542
F(000)	2728.0	8508.0
T [K]	150(2)	150(2)
λ (Mo Kα) [Å]	0.71073	0.71073
2θ (min., max.) [°]	4.224, 60.584	3.834, 50.054
Index range (h, k, l)	-23:23, -26:26, -27:27	-24:24, -25:25, -47:47

Total reflections	202033	335424
N_{ref}, N_{par}	29950, 1308	54501,3811
R(int)	0.1314	0.052
R_1 (I>2σ(I))	0.0584	0.0549
R_1(all data)	0.1166	0.0686
wR_2 (I>2σ(I))	0.1189	0.1362
wR_2 (all data)	0. 1493	0.1455
S	0.979	0.976
CCDC	2328826	2328827

$R_{int} = \Sigma|F_o^2 - F_o^2(\text{mean})| / \Sigma[F_o^2]$

$R_1 = \Sigma||F_o| - |F_c|| / \Sigma|F_o|$

$GOOF = S = \{\Sigma[w(F_o^2 - F_c^2)^2] / (N_{ref} - N_{par})\}^{1/2}$

$wR_2 = \{\Sigma[w(F_o^2 - F_c^2)^2] / \Sigma[w(F_o^2)^2]\}^{1/2}$

w = 1 / [σ^2(F_o^2) + (0.1200 * P)^2 +3000.00 * P], where P = (Max (F_o^2, 0) + 2 * F_c^2) / 3

Supplementary Table 2. BVS calculations and coordination numbers (CN) of Ce ions in the structure of Ce_{14}.

Atom	Ce^{III}	Ce^{IV}	CN
Ce1	**3.02**	2.65	9
Ce2	4.43	**3.90**	8
Ce3	4.44	**3.91**	8
Ce4	4.31	**3.79**	8
Ce5	4.68	**4.12**	8
Ce6	4.19	**3.69**	8
Ce7	**3.05**	2.68	10

Supplementary Table 3. BVS calculations, assignments, and CN for O atoms in the core of Ce_{14}.[a]

Atom	BVS	Assignment	CN
O3	1.95	O^{2-}	3
O4	2.04	O^{2-}	3
O8	2.03	O^{2-}	3
O10	1.94	O^{2-}	3
O11	1.18	OH^-	3
O7	1.89	O^{2-}	4
O9	1.93	O^{2-}	4
O13	1.93	O^{2-}	4

[a] For O atoms, BVS values in the ~1.8-2.0 and ~0.9-1.2 are attributed to non-protonation O^{2-} and single protonation OH^-, respectively.

Supplementary Table 4. Bond lengths of Ce-O (Ce-OH) in the structure of Ce_{14}. Parenthesis indicates errors.

Ce	O	Length /Å	Ce	O	Length /Å
Ce1	O7	2.419(5)	Ce4	O21	2.415(9)
Ce1	O11	2.464(5)	Ce4	O22	2.468(7)
Ce1	O1	2.476(5)	Ce4	O32	2.586(5)
Ce1	O12	2.489(8)	Ce5	O10	2.175(5)
Ce1	O28	2.536(6)	Ce5	O8	2.212(6)
Ce1	O31	2.565(7)	Ce5	O4	2.266(5)
Ce1	O33	2.574(6)	Ce5	O7	2.365(6)
Ce1	O25	2.735(7)	Ce5	O23	2.373(7)
Ce1	O1B^b	2.75(6)	Ce5	O24	2.428(6)
Ce1	O1A^a	2.926 (21)	Ce5	O25	2.459(6)
Ce2	O8	2.157(6)	Ce5	O1B^b	2.54 (4)
Ce2	O3	2.216(4)	Ce5	O1A^a	2.572(21)
Ce2	O13	2.296(5)	Ce6	O3	2.260(5)
Ce2	O7	2.308(6)	Ce6	O10	2.275(6)
Ce2	O5	2.367(8)	Ce6	O13	2.333(5)
Ce2	O14	2.392(4)	Ce6	O9	2.336(6)
Ce2	O15	2.514(6)	Ce6	O7	2.345(5)
Ce2	O32	2.581(5)	Ce6	O13	2.387(5)
Ce3	O3	2.201(6)	Ce6	O11	2.424(5)
Ce3	O4	2.201(6)	Ce6	O26	2.516(7)
Ce3	O8	2.270(5)	Ce7	O6	2.386(5)
Ce3	O16	2.355(6)	Ce7	O9	2.394(5)
Ce3	O9	2.361(5)	Ce7	O11	2.462(6)
Ce3	O17	2.434(5)	Ce7	O27	2.519(6)
Ce3	O18	2.448(6)	Ce7	O30	2.531(7)
Ce3	O19	2.487(6)	Ce7	O29	2.560(7)
Ce4	O4	2.164(5)	Ce7	O33	2.636(6)
Ce4	O10	2.235(4)	Ce7	O19	2.769(5)
Ce4	O9	2.312(6)	Ce7	O17	2.915(6)
Ce4	O13	2.330(6)	Ce7	O20	2.968(6)
Ce4	O20	2.379(5)	**Ce-O$_{(average)}$**		**2.440**

Supplementary Table 5. BVS calculations and CN of Ce atoms in the structure of Ce_{24C}.

Atom	Ce^{III}	Ce^{IV}	CN
Ce1	4.45	**3.91**	9
Ce2	4.22	**3.72**	8

Ce3	4.46	**3.92**	9
Ce4	4.41	**3.89**	8
Ce5	4.40	**3.86**	9
Ce6	4.72	**4.16**	8
Ce7	4.99	**4.39**	8
Ce8	4.50	**3.96**	9
Ce9	4.34	**3.81**	9
Ce10	4.75	**4.19**	8
Ce11	4.44	**3.90**	9
Ce12	**3.00**	2.63	9
Ce13	**3.15**	2.77	10
Ce14	4.45	**3.92**	8
Ce15	4.45	**3.91**	9
Ce16	4.30	**3.79**	8
Ce17	4.43	**3.89**	9
Ce18	4.21	**3.71**	8
Ce19	4.49	**3.95**	9
Ce20	4.37	**3.84**	9
Ce21	4.48	**3.94**	8
Ce22	4.33	**3.82**	8
Ce23	4.36	**3.83**	9
Ce24	4.25	**3.74**	9

Supplementary Table 6. BVS calculations, assignments, and CN for O atoms in the core of Ce_{24C}. [a]

Atom	BVS	Attribution	CN
O11	0.60	OH^-	3
O19	1.94	O^{2-}	3
O21	1.38	OH^-	3
O27	2.00	O^{2-}	3
O29	1.25	OH^-	3
O30	1.95	O^{2-}	3
O32	1.98	O^{2-}	3
O34	1.61	O^{2-}	3
O39	2.03	O^{2-}	3
O68	1.88	O^{2-}	3
O70	1.98	O^{2-}	3
O72	1.93	O^{2-}	3
O75	1.96	O^{2-}	3
O77	1.77	O^{2-}	3
O78	1.44	OH^-	3
O82	1.84	O^{2-}	3
O89	1.26	OH^-	3
O13	2.12	O^{2-}	4
O14	2.11	O^{2-}	4
O15	2.19	O^{2-}	4
O16	2.16	O^{2-}	4

O17	2.06	O^{2-}	4
O18	1.79	O^{2-}	4
O24	2.06	O^{2-}	4
O25	2.13	O^{2-}	4
O26	2.10	O^{2-}	4
O31	1.92	O^{2-}	4
O64	2.18	O^{2-}	4
O65	2.17	O^{2-}	4
O66	2.12	O^{2-}	4
O67	2.08	O^{2-}	4
O71	1.75	O^{2-}	4
O76	1.91	O^{2-}	4
O109	0.70	OH^-	4
O110	0.67	OH^-	4
O111	0.69	OH^-	4

Supplementary Table 7. Bond lengths of Ce-O (Ce-OH) in the structure of Ce_{24C}. Parenthesis indicates errors.

Ce	O	Length /Å	Ce	O	Length /Å
Ce1	O19	2.197(7)	Ce12	O109	2.801(6)
Ce1	O13	2.257(5)	Ce13	O64	2.386(5)
Ce1	O16	2.298(7)	Ce13	O65	2.388(7)
Ce1	O21	2.328(5)	Ce13	O73	2.47(1)
Ce1	O12	2.403(9)	Ce13	O63	2.51(1)
Ce1	O22	2.406(8)	Ce13	O74	2.517(7)
Ce1	O59	2.534(7)	Ce13	O107	2.550(7)
Ce1	O11	2.648(6)	Ce13	O99	2.68(1)
Ce1	O47	2.665(6)	Ce13	O69	2.70(1)
Ce2	O31	2.295(7)	Ce13	O110	2.825(8)
Ce2	O30	2.307(7)	Ce13	O111	2.866(6)
Ce2	O19	2.310(6)	Ce14	O64	2.308(6)
Ce2	O32	2.354(5)	Ce14	O65	2.311(5)
Ce2	O14	2.376(5)	Ce14	O25	2.313(5)
Ce2	O13	2.378(7)	Ce14	O66	2.316(5)
Ce2	O18	2.384(5)	Ce14	O67	2.320(8)
Ce2	O33	2.40(1)	Ce14	O26	2.330(8)
Ce3	O30	2.193(5)	Ce14	O76	2.359(6)
Ce3	O34	2.258(5)	Ce14	O76	2.384(5)
Ce3	O14	2.270(7)	Ce15	O72	2.206(7)
Ce3	O15	2.297(7)	Ce15	O65	2.251(5)
Ce3	O35	2.41(1)	Ce15	O66	2.276(5)
Ce3	O36	2.421(7)	Ce15	O78	2.318(9)

Ce3	O37	2.528(6)	Ce15	O79	2.380(7)
Ce3	O55	2.684(6)	Ce15	O80	2.470(7)
Ce3	O109	2.753(6)	Ce15	O74	2.529(7)
Ce4	O17	2.302(5)	Ce15	O100	2.614(8)
Ce4	O16	2.304(5)	Ce15	O111	2.705(7)
Ce4	O15	2.311(7)	Ce16	O76	2.296(5)
Ce4	O24	2.337(5)	Ce16	O75	2.311(7)
Ce4	O14	2.338(7)	Ce16	O68	2.315(8)
Ce4	O13	2.341(7)	Ce16	O72	2.319(6)
Ce4	O31	2.356(5)	Ce16	O66	2.354(7)
Ce4	O31	2.375(7)	Ce16	O25	2.357(5)
Ce5	O39	2.150(7)	Ce16	O71	2.396(8)
Ce5	O16	2.255(5)	Ce16	O81	2.398(7)
Ce5	O17	2.355(5)	Ce17	O82	2.203(7)
Ce5	O21	2.366(7)	Ce17	O68	2.223(5)
Ce5	O40	2.408(7)	Ce17	O25	2.276(8)
Ce5	O41	2.452(6)	Ce17	O67	2.292(5)
Ce5	O62	2.551(9)	Ce17	O83	2.435(8)
Ce5	O57	2.572(8)	Ce17	O84	2.482(9)
Ce5	O109	2.679(7)	Ce17	O106	2.560(8)
Ce6	O27	2.272(7)	Ce17	O102	2.592(8)
Ce6	O39	2.303(6)	Ce17	O110	2.788(7)
Ce6	O18	2.322(7)	Ce18	O70	2.298(6)
Ce6	O31	2.327(5)	Ce18	O76	2.322(8)
Ce6	O24	2.347(5)	Ce18	O67	2.335(7)
Ce6	O17	2.370(7)	Ce18	O77	2.337(5)
Ce6	O1B^b	2.40(5)	Ce18	O82	2.340(5)
Ce6	O29	2.409(7)	Ce18	O26	2.377(5)
Ce6	O1A^a	2.428(36)	Ce18	O71	2.391(5)
Ce7	O19	2.196(5)	Ce18	O42	2.41(1)
Ce7	O39	2.204(7)	Ce19	O68	2.205(7)
Ce7	O43A^b	2.32(3)	Ce19	O82	2.233(5)
Ce7	O43^a	2.361(26)	Ce19	O26	2.251(5)
Ce7	O18	2.366(6)	Ce19	O66	2.279(8)
Ce7	O44	2.39(1)	Ce19	O86	2.409(6)
Ce7	O21	2.387(8)	Ce19	O87	2.483(8)
Ce7	O20	2.411(8)	Ce19	O88	2.534(6)
Ce7	O45	2.422 (7)	Ce19	O80	2.635(5)
Ce8	O32	2.173(7)	Ce19	O111	2.787(6)
Ce8	O24	2.242(7)	Ce20	O70	2.208(5)
Ce8	O13	2.259(5)	Ce20	O64	2.284(7)
Ce8	O29	2.382(6)	Ce20	O26	2.290(6)

Ce8	O46	2.435(6)	Ce20	O89	2.353(6)
Ce8	O47	2.440(8)	Ce20	O69	2.417(6)
Ce8	O60	2.539(7)	Ce20	O90	2.419(9)
Ce8	O49	2.580(7)	Ce20	O105	2.522(7)
Ce8	O11	2.636(7)	Ce20	O87	2.625(6)
Ce9	O27	2.180(6)	Ce20	O111	2.631(6)
Ce9	O15	2.246(5)	Ce21	O70	2.171(5)
Ce9	O34	2.320(8)	Ce21	O75	2.201(9)
Ce9	O24	2.352(7)	Ce21	O91	2.294(6)
Ce9	O48	2.363(8)	Ce21	O71	2.351(7)
Ce9	O49	2.508(7)	Ce21	O92	2.409(9)
Ce9	O56	2.567(7)	Ce21	O93	2.429(9)
Ce9	O61	2.605(7)	Ce21	O89	2.437(7)
Ce9	O11	2.737(7)	Ce21	O94	2.439(7)
Ce10	O30	2.198(5)	Ce22	O72	2.186(8)
Ce10	O27	2.210(8)	Ce22	O77	2.248(6)
Ce10	O34	2.335(6)	Ce22	O95	2.32(1)
Ce10	O50	2.360(6)	Ce22	O71	2.349(5)
Ce10	O18	2.380(7)	Ce22	O78	2.372(8)
Ce10	O51A^b	2.40(2)	Ce22	O97	2.432(9)
Ce10	O52	2.42(1)	Ce22	O96	2.433(6)
Ce10	O53	2.472(7)	Ce22	O98	2.462(7)
Ce10	O51^a	2.474(34)	Ce23	O75	2.179(7)
Ce11	O32	2.178(5)	Ce23	O64	2.263(5)
Ce11	O17	2.247(7)	Ce23	O25	2.281(7)
Ce11	O14	2.260(5)	Ce23	O89	2.395(8)
Ce11	O29	2.399(7)	Ce23	O101	2.400(8)
Ce11	O54	2.402(6)	Ce23	O102	2.486(7)
Ce11	O55	2.448(8)	Ce23	O107	2.544(6)
Ce11	O58	2.535(6)	Ce23	O108	2.560(8)
Ce11	O41	2.590(5)	Ce23	O110	2.710(9)
Ce11	O109	2.710(6)	Ce24	O77	2.215(7)
Ce12	O15	2.382(5)	Ce24	O65	2.301(7)
Ce12	O16	2.399(7)	Ce24	O67	2.306(5)
Ce12	O38	2.49(1)	Ce24	O78	2.345(6)
Ce12	O23	2.502(9)	Ce24	O103	2.408(9)
Ce12	O56	2.512(7)	Ce24	O99	2.450(7)
Ce12	O57	2.524(6)	Ce24	O104	2.532(9)
Ce12	O22	2.66(1)	Ce24	O84	2.615(7)
Ce12	O36	2.70(1)	Ce24	O110	2.676(8)
			**Ce-O$_{(average)}$**		**2.405**

Supplementary Table 8. Main peaks and corresponding assignments (Durgaprasad et al., 1971, Gu and Soucek, 2007, Lewandowski and Baranska, 1986) in the IR spectra for Ce_{14} and Ce_{24C} crystals.

IR peaks in Ce_{14} (cm^{-1})	IR peaks in Ce_{24C} (cm^{-1})	Assignment
465	486	Ce-O stretching
673	675	$\delta(OCN)_{DMF}$
713	716	$\gamma(COO)_{PhCOO}$
847	849	$\nu(CN)_{DMF}$
937	939	$\gamma(CH)_{PhCOO}$
1024	1026	$\beta(CH)_{PhCOO}$
1068	1068	$\gamma(CH_3)_{DMF}+\nu(CN)_{DMF}$
1255	1254	$\nu(CN)_{DMF}$
1396	1396	$\nu(COO)_{PhCOO}$
1446	1448	$\nu(C{=}C)_{PhCOO}$
1491	1492	$\nu(C{=}C)_{PhCOO}$
1545	1537	$\nu(COO)_{PhCOO}$
1595	1593	$\nu(C{=}C)_{PhCOO}$
1666	1668	$\nu(CO)_{DMF}$
3061	3061	$\nu(CH)_{PhCOO}$

Supplementary Table 9. Fitting parameters from the Guinier analyses of SAXS data for DMF solutions of Precipitate-1 and Precipitate-2.

**Precipitate-1**	$q_{min}R_g$	$q_{max}R_g$	**Precipitate-2**	$q_{min}R_g$	$q_{max}R_g$
**2h**	0.15	0.50	**2h**	0.56	0.99
**12h**	0.18	0.51	**12h**	0.56	0.97
**2d**	0.20	0.58	**2d**	0.49	0.90
**4d**	0.21	0.73	**4d**	0.52	0.93
**8d**	0.22	0.75	**8d**	0.48	0.89

References

Durgaprasad, G., Sathyana & Patel, C. C. (1971). Infrared Spectra and Normal Vibrations of N,N-dimethylformamide and N,N-dimethylthioformamide. *Bull. Chem. Soc. Jpn.* 44, 316-322.

Gu, H. & Soucek, M. D. (2007). Preparation and Characterization of Monodisperse Cerium Oxide Nanoparticles in Hydrocarbon Solvents. *Chem. Mater.* 19, 1103-1110.

Lewandowski, W. & Baranska, H. (1986). Vibrational and Electronic Spectroscopic Study of Lanthanides and Effect of Sodium on the Aromatic System of Benzoic-acid. *J. Raman Spectrosc.* 17, 17-22.
